# Supplementary material for: Exploring the Participant-Related Determinants of Simulator Sickness in a Physical Motion Car Rollover Simulation as Measured by the Simulator Sickness Questionnaire
Source: Int J Environ Res Public Health. 2020 Sep 26;17(19):7044. doi: 10.3390/ijerph17197044 (PMC7579369; doi:10.3390/ijerph17197044)
Supplement: Supplementary file 1 [file ijerph-17-07044-s001.pdf]

Simulator sickness symptoms[1]

**General discomfort**

**Fatigue**

Boredom

Drowsiness

**Headache**

**Eyestrain**

**Difficulty focusing**

**Increased salivation**

Decreased salivation

**Sweating**

**Nausea**

**Difficulty concentrating**

Depression

**Fullness of head**

**Blurred vision**

**Dizziness (eyes open)**

**Dizziness (eyes closed)**

**Vertigo**

Visual flashbacks

Faintness

Awareness of breathing

**Stomach awareness**

Decreased appetite

Increased appetite

Desire to move bowels

Confusion

**Burping**

Vomiting

---

[1] These symptoms are listed by Kennedy et al. (see: Kennedy, R.S.; Lane, N.E.; Berbaum, K.S.; Lilienthal, M.G. Simulator Sickness Questionnaire: An enhanced method for quantifying simulator sickness. *Int. J. Aviat. Psychol.* 1993, 3, 203–220, doi:10.1207/s15327108ijap0303_3) as a basis for developing the SSQ. Our study participants were asked to assess their severity on the *none-slight-moderate-severe* scale, but only 16 of the symptoms (in **bold**), regarded by Kennedy et al. as the core symptoms characterising simulator sickness, were scored and used in statistical analyses. We used the Polish version of the questionnaire translated by Biernacki et al. (see: Biernacki, M.P.; Kennedy, R.S.; Dziuda, Ł. Zjawisko choroby symulatorowej oraz jej pomiar na przykładzie kwestionariusza do badania choroby symulatorowej – SSQ. *Med. Pr.* 2016, 67, 545–555, doi:10.13075/mp.5893.00512).
